# Struggling on My Own: A Cognitive Perspective on Frequent Attenders' Conception of Life and Their Interaction with the Healthcare System

**DOI:** 10.1155/2013/580175

**Published:** 2013-04-16

**Authors:** Lena Wiklund-Gustin

**Affiliations:** School of Health, Care and Social Welfare, Mälardalen University, Box 883, 721 23 Västerås, Sweden

## Abstract

Different studies reveal that a large percentage of people frequently attending healthcare not only suffer from diffuse somatic symptoms but also from psychological distress and difficulties in dealing with everyday life. Even though they are not always diagnosed with psychiatric disease, questions arise about their mental health. The study aims at describing frequent attenders' conceptions of life, and as a result their health, from a cognitive perspective. A qualitative content analysis of in-depth interviews was carried out with nine service users in primary healthcare. The findings reveal that participants experience themselves as inadequate and as being a burden for others, by whom they experience rejection, in different ways. In order to take part in community with others the person develops compensatory strategies that aim at concealing their inadequacies, thus also preventing them from sharing their suffering with others. The consequence is that the persons become even more alienated as they start to relate to others through a façade and furthermore are unable to either improve their health or obtain adequate care. It can be concluded that these patients need to be taken seriously in order to prevent further psychological suffering.

## 1. Introduction

Frequent attenders appear to be a group troubling the healthcare system in different ways. Firstly, it is a group that consumes much of the resources. Various studies from around the world reveal that between 2% and 10% of the population accounts for 15–40% of the costs [1–4]. Secondly, they trouble healthcare staff [5], as many of the patients present diffuse symptoms like pain and dizziness which may be hard to diagnose [6].

The group has been defined in different ways, making it hard to compare results and define the group [7]. There is also a difference between frequent attenders that attend for a diagnosed chronic disease and those suffering from vague symptoms where their general practitioners are unable to diagnose them [6]. Even though the group is not clearly defined, numerous efforts have been made to describe it, using quantitative methods that focus on quality of life [8], social factors [9–11], and comorbidity with psychiatric disease [8, 12–14]. Furthermore, frequent use of health services also appears to be associated with a low sense of coherence and low internal locus of control [15–17].

Schilte et al. [18] conclude that 80% perceive their childhood as troublesome. The authors also claim that to understand the frequent attender, one must approach the problems from a biopsychosocial perspective. In their study, they identify physiological as well as psychological and social risk factors that contribute to the problems. Chronic disease is supposed to play a major role as well as experiences of pain. However, the person's perception of whether the symptoms limit his/her life, and if the symptoms result in thoughts that give rise to feelings of worry and anxiety appear to be central issues, as are the person's illness perceptions [4].

These results could be understood in two ways: either as if the frequent attenders have psychiatric problems from the beginning and are somatising them, or the patients develop psychiatric symptoms as a consequence of unexplainable illness experiences. Whatever the reason, there is a group of patients suffering from physical as well as psychological problems, and there are also some studies pointing to a high degree of unmet health needs [19] as well as the importance of understanding the patient in relation to his/her context, not only focusing on symptoms [20]. Furthermore, qualitative research reports that these patients constantly strive to be and become healthy in order to please others, and that as a consequence they hesitate to attend until they experience suffering as unbearable or life threatening [21, 22]. Thus, patients' perspectives differ from health professionals who view of them as troublesome, having a “wrong attitude” and “undesired behavioural patterns” [23]. All this calls for further exploration about how these persons construct their view of their life and health, in order to understand their motives for attendance. 

*Theoretical Perspective.* Cognitive behavioural therapy (CBT) has recently been suggested as a useful framework for understanding frequent attenders' health seeking behaviours and also for treatment [3]. This motivated an adoption of a CBT-framework in order to guide the formulation of research questions as well as interpretation of data. The theoretical perspective is based on Beck's cognitive model for understanding and treating psychological distress [24, 25]. From this perspective, persons' conceptions of life relate to a cognitive process where meanings are ascribed to experiences. On the most fundamental level, meaning is constructed in relation to self, the environmental context/the world and the future, which constitutes the cognitive triad. Thus, the cognitive triad relates to core beliefs that give rise to conditional assumptions, guiding the person's orientation and performances in life [25]. Compensatory strategies grow out of these assumptions and beliefs in order to shield the person from suffering related to negative core beliefs and conditional assumptions. However, life situations that challenge core beliefs as well as conditional beliefs, will give rise to automatic thoughts, that is, words or images that pass the person's mind on a more superficial level of cognition. As the construction of meaning arises in interactions with others, the attachment theory is incorporated in the theoretical frame in order to promote interpretation of findings.

*Aim of the Study. *This study aims at describing frequent attenders' conceptions of life and of their interaction with the healthcare system from a cognitive perspective. The research thus tries to answer the following questions. (1) What are the participant's beliefs about their life situation? (2) What compensatory strategies do they use in order to deal with life? 

## 2. Methods

### 2.1. Participants

Before starting the study, it was approved by a local research committee. Data were obtained by means of interviews with nine persons, five women and four men. The participants were recruited among persons who were also offered the chance of taking part in a CBT-group aiming at enhancing their ability to cope with their present situation. This also makes it possible to evaluate experiences of participating in the group programme. From the beginning, ten persons were recruited, but one dropped out due to personal problems. As this occurred at a time when the process of recruiting group participants had ended and nine persons are in line with Kvale's (1996) recommendations for this kind of interview, we did not ask anybody else. The subjects had been in contact with their GPs five times or more during the past year which was the inclusion criteria for the CBT-group and in line with Andersson et al.'s [1] definition of the group, which is commonly used in Sweden. An overview of participants is presented in Table 1.

Participants were first informed about the research by a person working at a primary healthcare centre, and when they expressed interest in participating in the study, they were given further information orally as well as in writing from the researcher. Thus, taking part in research was based on informed consent, and the participants also knew that taking part in the study would not in any way influence their treatment. In order to respect individuals' rights and dignity, participation was voluntary and could be interrupted at any time, and confidentiality was guaranteed by altering data so that no participants could be recognised in the report. 

### 2.2. Collection of Data

The interviews lasted between one and two and a half hours, with an average of just under two hours and were carried out before going into CBT-group treatment. Interviews started with an open question in which subjects were asked to describe how they viewed their current life situation. The answers were followed by further questions that arose from the subject's description and focused their thoughts, feelings, and actions in relation to their current situation as well as what earlier experiences in life they perceived as important in relation to today's health and wellbeing. 

### 2.3. Analysis and Interpretation of Data

The interviews were transcribed verbatim, and the transcripts constituted the empirical material for qualitative content analysis [26]. The text was read repeatedly to obtain a sense of the whole. During this process two concept areas were identified: beliefs and compensatory strategies. In the next steps, meaning units in the text were marked, condensed, and abstracted into codes, subcategories, categories, and themes. At this point, the findings were reflected upon in relation to the theoretical perspective that thus provided a framework for interpretations during the last step. 

## 3. Findings

The presentation of findings arises from a reflective process moving between methodological steps and the theoretical frame. The first concept area, participants' beliefs, relates to the first research question and is formulated as three categories related to the cognitive triad, while the other concept area reflects one category about compensatory strategies. Subcategories in the text are written in *italics*.

### 3.1. Beliefs about Self—“I'm Inadequate”

The main category relating to the aspect of the cognitive triad describing the participant's conception of self as not being good enough as a person. In order to compensate for their perceived shortcomings, conditional assumptions and rules guide their way of behaving in a way they perceive will render them the appreciation of others. The participants also describe that this is not a new experience for them; rather it is something that they had learned in early life. 

One conditional assumption that the subjects put forth is *I must always perform the very best and preferably a little bit more than that.* This assumption also forms the first subcategory. This has been learned from early age, and the participants describe how they had to be of use at home before going out to play or even doing their homework. Thus, they have learned the importance of being a person that must perform well at home as well as at work, to receive appreciation.

*My dad had been ill, and my mother had arthritis but she was working anyway. Cleaning at others and things like that. So when I came home from school I had to light the fire at home and do the cooking. Such things. And nobody ever helped me with my homework, but I was still expected to learn everything.*

Experiences like this have been guiding the lives of the participants and characterised the person's perception of the present and how to handle it. The participants express an apprehension that they must perform very well to receive appreciation from others. This assumption about performance contributes to the participant's suffering and their experience of failure in their interaction with the healthcare system.

Another subcategory, reflecting on the participants' view of self, is the assumption *I must stay healthy and not cause any trouble*. Participants believe that they must be healthy, preferably merry people who do not cause any trouble for others. When succeeding in this effort, they will be able to preserve their relations with other people. The participants describe a fear of failing, as they believe that doing this will make others reject them. Those participants who have been ill in early life describe how they learned to hide away their feelings of discomfort, illness, or disappointment.

*Well I'm grown up with that, that I should not complain (when feeling ill). I grew up with my grandparents, and learned that I had to be nice and keep quiet. And if I complained, they just gave me fish-liver oil and sent me off to school.*

If they complained they were ignored, or even punished for doing so, even though the punishments were often subtle. Thus, the participants tell about being ignored and left alone rather than physically abused when ill. Having these experiences results in an apprehension that there is no use asking for help when feeling ill unless it is really serious.

Another subcategory reflects the assumption that *I must not lose control*. By controlling the situation, for example, by being nice, helping others, working hard, ignoring one's own needs, and soon, one is able to compensate for one's own perceived shortcomings.

*I do my share even though it has a price and becomes too much. Because I want to stay in control. If I don't know what's happening around me and I lose control when I get frightened.*

When acting in a specific way, it is possible to predict other people's reactions as well as getting appreciation for what has been established. However, the sense of control is a very fragile one, as it is not grounded inside the person but related to performance. By doing one's best, showing a healthy and merry façade, it is possible to predict the reaction of others, which gives rise to a fragile sense of security that enables the person to take part in social interactions. 

When illness prevents the person from doing what is perceived as expected, thoughts telling them *I am a burden for others* will appear. This is evident among all participants but appears to be even more troublesome for participants living together with others, as they experience that they are causing their loved ones trouble. The participants start to view themselves as burdens not only in daily interactions with family, colleagues, and friends but also as burdens for society. They describe feelings of insufficiency constantly nagging their self-esteem, promoting the experience of themselves as a burden. For example, the participant quoted below describes his feelings of being a burden because of his symptoms, but also his fear of becoming a burden if he receives proper treatment, as he believes that he would then hinder someone else, more entitled to care, from receiving help.

*I feel more or less like a huge burden for society. If there were any treatment for this that they could give me it would be fine. But if so, I would ponder whether I might push anybody else aside, who is in greater need than I am.*

*(Researcher) So what you are saying is that if you do not receive treatment you will go on being a burden, and if you receive it you will be a burden in another way?*

*Yes, I think I'm selfish if I claim this help. Or maybe it is because of my belief that one should not involve other people (one should manage on one's own). Now I feel as I have to beg to get anything and involve others. So I prefer putting it off until tomorrow.*

*(Researcher) You don't want to cause any trouble for others, is that right?*

*Yes. Because my own thoughts when I go there are that they think “Oh now, here he comes again”. So I postpone it as much as possible.*

In this quotation too, the conception that one is not good enough unless one is not healthy and does one's share underpins the discourse. To ask for care is perceived in terms of selfishness. It could also be interpreted as a conception of other people as more important than oneself. 

### 3.2. Beliefs about the Environmental Context—“There Is No Place for Me in the World”

The meaning given to the environmental context, the shared human world, is constructed around interactions with others. Human beings are, as Bowlby [27] points out, predisposed from birth to interact with others. Attachment is perceived as a basic need for human survival; therefore, the person constantly strives to act in a way that will establish and/or preserve bonds to others. If one, as mentioned above, believes that one is adequate it will be necessary to focus on how other people appear to react when interacting with them in order to be able to adjust to a demanding world, trying to fit into it. Thus, participants describe how they “bite their tongues” as long as possible, avoiding asking for help and showing weakness unless it is perceived as very urgent. When they ask for help, their experience is that they are rejected in different ways; experiences that will nourish the beliefs about themselves as inadequate.

A problem recurrent in all the participants' narratives is the experience of being put aside and neglected when attending for care. Thus, *others neglect and overrun me* forms the first subcategory in relation to the category that deals with beliefs about having no place in the world. The narratives disclose that sometimes one has not even been able to obtain an appointment with the GP.

*Being sent home like that, when you know that there is something wrong affects me and I start asking myself: Am I imagining that I'm in pain?*

Thus, the participants' experiences are marked by a sense of not being taken seriously. Rather, they experience that the staff mistrust and question their causes for attending and therefore put them off. This will nourish their conception that they must be healthy to be treated seriously, and/or that other people's opinions are more trustworthy than their own, as their own description of the situation is not taken into account. Instead, it is others who tell them the truth about how they really feel, or at least ought to feel.

Another subcategory could be expressed as *other people do not understand me*, especially not the healthcare staff that are supposed to help them. Similar thoughts are described in relation to people in their daily environment.

*I have had a pain in my neck for years now, and tried to get something done about that. But that isn't easy, you know, as they brush me off, or talk me down in one way or another. Telling me to go home and stretch and then everything will be OK. Well, it will not, and finally I have become more or less pissed off about the whole system.*

*(Researcher): What is it that makes you become that angry?*

*Well basically, they don't take me seriously. They do understand the words I'm saying, but they don't understand the situation and that it is serious. That seems to be impossible. I wish I had something that they could see with their own eyes, and then I think it might be possible to be listened to, to make them believe in me and receive some help. If I came there with a huge wound they could not send me away, could they? I just don't know what to do to make them believe in me. I just don't have any more strength to argue.*

This too will nourish the patients' beliefs that they are inadequate and not significant as persons. Thus, the negative spirals will go on, the person believes that he/she has to manage on his/her own and that he/she is not entitled to care. 

Underlying the former assumptions about others as neglecting and misunderstanding is an assumption that *other people mistrust me.* The participants strive against a suspicion that other people mistrust and question them and their motives. An experience that is described often is that other people, healthcare staff as well as friends and colleagues, believe that they are actually not sick, and that they attend the healthcare centre just to get advantages such as early retirement or a doctor's certificate so that they do not have to work. In other words, their automatic thought is that others do not only mistrust them, they think they are lazy and are trying to avoid work and other duties.

*She didn't believe there was anything wrong with me. “I can't see anything”, she said, “There is nothing wrong with you. You just don't want to work”. I always heard those kinds of things. But it isn't true.*

As the participant's life is guided by a rule that one has to perform one's very best and do one's share, the suspicion that other people think they are trying to escape their responsibility is devastating, as it contains a threat against the person's values and perceptions of self. This experience of being questioned and doubted is humiliating and increases the person's suffering even more, as it is not only the person's behaviour that is questioned but also his/her being.

### 3.3. Beliefs about the Future—“You Never Know What Will Happen”

The third aspect of the cognitive triad is related to the beliefs the person has about the future. Overall, the participant's view their future as impossible to predict, it could turn out in any direction. Feelings of hope are paired with despair. An optimistic view of whatever the future might bring could be a motivating force to change, while a negative or pessimistic conception is often linked to feelings of powerlessness and depression. The subcategories arising in relation to this aspect are both positive which reflects a sense of hope, and negative, promoting the participants' feelings of inadequacy.

The first subcategory related to beliefs about the future is* something might be done that changes everything*. However, rooted in the participants' experiences of not being listened to, this subcategory thus reflects a glimpse of hope. As the participant believes that the GP has not really understood the problem; there is hope that when this understanding appears, there will also be an appropriate treatment for the patient's problem.

*As nobody could give me a proper diagnosis, nobody is able to rehabilitate, or come to any decisions or anything that makes sense. But I don't think that everything has been done yet. One day they will find out, so I will not give up.*

In this case, despair will give the person strength to go on; the person truly believes that something will eventually happen that changes everything. When asked why they keep on attending even though they experience so many disappointments and lack of understanding some of the participants answer “There is nothing else to do, and I can't stop hoping.”

Other subcategories reflect more negative conceptions and an exhausted feeling, for example, *I will never receive any help. *The participants' experience themselves as being shuffled around between different GPs never obtaining any answers as to what is wrong or what could be done.

*I have accepted anything lately, just to have them to do something I accept whatever they suggest. But nobody finishes anything, they just say, “I'm not the person who should deal with this”. And then, of course, I think that they will pass me on to an expert or something, someone who really can manage the problems. But that's not how it is; they just don't want to do anything to help. *

Even though the participant intellectually understands that some of the problems, such as a lack of continuity, are organisational and out of their own control, they emotionally experience the situation as a personal failure. They end up blaming themselves not only for being a burden but also for not being able to communicate their suffering to others in a proper way.

Another subcategory related to the future, *I don't dare to do this,* concerns fear. This emotion restricts life, as the fear of symptoms often makes the patients think that desired activities might be interrupted by pain or other distressing symptoms. Having to interrupt planned activities will serve as a reminder that one failed to accomplish what one ought to be able to do. As success is more or less a proof of one's value, failure will mean not only that one has failed with one activity but rather that one is a failure as a person.

*We used to go to Finland on vacation, which is about one thousand kilometres if you go by car. But now I'm dreading that trip. Should I go there? Could I go there? What will happen if the pain comes, if I must go to hospital? And how will it be, for me? For my family? Will I spoil their pleasure? Maybe they think I'm nagging if I complain? I have to balance all those things before deciding anything.*

### 3.4. Compensatory Strategies to Deal with Suffering—“I Must Not Show My Weakness”

To understand the suffering of the frequent attender one must also focus on the person's attempt to deal with it. Compensatory strategies are the strategies the person uses to avoid confrontation with the emotional pain of not being loved as the person really is and of not being allowed to take part in an unconditional communion with others. *To do one's share *thus is a compensatory strategy relating to a basic belief of being a burden. *Avoiding attending* the healthcare unit is another—the participants postpone their consultation until they experience suffering as unbearable. They try to handle the situation on their own as much as possible; they go to work even when their health would be better if they stayed at home.

As they are struggling with daily life as well as with being able to work to avoid being a burden, they also struggle with their symptoms and avoid asking for help from the healthcare system. The participants' opinion is that they do not attend unnecessarily; rather, they strive to manage by trying to *ignore the problems*, by distracting themselves from them. 

Another strategy, coherent with the conditional belief that one has to be healthy and merry to stay in communion with others, is to conceal their alignment for others. In other words, one strives to fit in by distancing from symptoms and *show a positive façade*.

*Well, I put on a smile anyway.*

*(Researcher): Why do you do that?*

*Partly I don't think that people want to see me with the corner of my mouth pointing down, partly I'm so tired of trying to explain my situation to people who really don't understand anyway.*

Behind the façade dwells an experience of resignation. There is no use showing how one really feels as nobody will care or understand. It seems more appropriate, and easier, to put on a happy face and hide behind that. However, this strategy does not only temporarily relieve suffering but also alienates the participant even further from others as it becomes almost impossible for others, to recognise pain and suffering.

*Well, I don't show I'm in pain and feeling ill. I rarely do that. Because it makes it easier if one can laugh at the misery and say “Oh dear, here is the old woman coming again, biting me here and there”, and laugh. And because of that people don't believe me. “One doctor told me “You can't be ill, looking that happy”.*

Not experiencing oneself as understood by others increases suffering, as it nourishes the conception that one is not worth the concerns of others. Thus, one carries on hiding the problems from others, hiding behind the façade and struggling with everyday life.

*Well, I manage. One mustn't think about the pain. But sometimes I keep on going so much, that when I come home in the evening I'm worn out. Often I've been in so much pain that I hardly manage to make my way home from work. But I've been of use, helping others.*

This struggling helps the person to live according to his/her basic conceptions and shelters him/her from the emotional pain of shame and shortcomings. Even painful physical symptoms are perceived as easier to bear than psychological suffering. Living according to those rules of how life ought to be lived, becomes a way of dealing with life. On the other hand, acting against them involves a risk of being excluded from the community with others, which according to Bowlby [27] is a big threat.

### 3.5. Thematic Understanding—“Struggling on My Own”

The theme underpinning the discourse describes participants' conceptions about their life situation and their interactions with the healthcare system as a struggle on their own. Participants conceive themselves as inadequate and more or less abandoned by other people and believe that they must manage on their own and/or adjust to others. Underlying the discourse is the person's struggle not only for getting a proper diagnosis and treatment in order to regain their health but also to be loved, appreciated, and in contact with others. Compensatory strategies should be understood in the light of people continually striving to conceal their shortcomings and relate to others.

Unfortunately, participants do not succeed very well in this lonely struggle. The constant stress of performing at their very best also means that they are not listening to bodily signals until they are almost unable to endure their health problems. The façade, aiming at sheltering the patient, contributes to further suffering rather than helping it because it (a) hinders the person to be him/herself in relation to others, and (b) the “happy face” that comes with the façade gives people in the environment the false impression that the patient's health problems are not serious ones. Thus, the strategies aiming to alleviate suffering in the long term will increase it instead, as they keep the person struggling alone, unable to ask and receive care based on a holistic understanding of the situation.

## 4. Discussion

To what extent could we learn something from these findings? Are these categories and subcategories too general, something that almost everyone could identify with? In a way, yes, this is true. In the western world the culture strongly focuses on fame and success, and furthermore, we are often told that people who are sick are a tremendous cost for society. Thus, it is easy to understand that this kind of conception is common and that it is nourished in the media. On the other hand, even if most people could recognise the thoughts of the participants in their lives, they are not ruled by them to the same extent as the frequent attender. As long as a person can distinguish his/her thoughts and outer as well as bodily experiences in a proper way, it does not create any greater problems. But when stress overwhelms a vulnerable person, the problems described in this study might arise, causing major personal suffering for the individual and great costs for society.

The point of departure for a discussion on whether the research results are useful and trustworthy is a view of nature as not being uniform. Thus, all frequent attenders do not conceive of life and their interaction with the health care system in exactly the same way as the participants in this study, nor do they have exactly the same compensatory strategies. However, the result may be helpful in understanding the suffering patient. In other words, if the study contributes knowledge that facilitates caregivers' understanding of the frequent attending patient, and if the caregiver can make use of this knowledge when interacting with the patient, one can claim that the results have been generalized. Thus, generalizability is based on transformation of meaning, not related to numbers [28]. In other qualitative methods, such as the grounded theory, researchers often talk about saturation as a sign of having included enough participants in a study to cover the field. In these kinds of studies, participants are often recruited consecutively until no new categories arise during analysis. In this study, all participants were recruited before starting the interviews, in order to coordinate the interviews with the timetable for the CBT-programme. Even though saturation is a concept rarely used within a hermeneutic tradition, it could be concluded that the same category was repeated in the different interviews and that data could be viewed as being sutured. Variations were rather related to how categories were expressed than to content. For example, there were different ways to ignore the problem, but a common thread of meaning was that participants strived to distance themselves from suffering by ignoring their afflictions.

Another question concerns reliability and validity. How trustworthy are the results? Validity in qualitative research concerns the question of whether or not the researcher has been able to extract and mediate the meaning of the data in a way that makes sense. Validity thus is related to whether the result makes it easier to get to know and understand the phenomena [29].

Closely related to this is the question about reliability, or whether data are trustworthy. The point here is not whether the participants are describing an objective truth. The patients' narratives are based upon their conceptions of their life or of a specific situation. Another person, for example, their GP might perceive and describe it in a totally different way. However, the aim of this study was not to describe “how it is” but to describe how the frequent attender conceives the situation; that is, a subjective perspective of the participant's life-world. That is in line with the theoretical perspective. The cognitive view of how the person functions mentally is that we live in a world based on subjective interpretations. It is the patient's subjective beliefs and assumptions that the staff deal with when interacting with the patient. The result thus should be looked upon from that perspective, and validity thus becomes closely linked with generalizability. Kvale [30] puts forth communicative and pragmatic validity as a criterion for qualitative research. Research findings are valid, and possible to generalise, when they are communicated to the community in a way that makes sense, that is, when they become true in general practice by guiding the practitioner's thinking. 

The result is in line with earlier research based on quantitative approaches. Several studies [8, 31–33] claim that there is a psychological distress among frequent attenders, and anxiety and depressive symptoms appear to be common. However, in those studies little or no interest has been paid to the cognitive content in depressive or anxiety-full thoughts within this group of patients. 

This study thus contributes new knowledge to that area by putting the focus of the cognitive content on core beliefs as well as conditional assumptions. The study suggests there is a link between the patients' earlier history, their symptoms, and their health-seeking behaviours. Thus, the study also confirms the importance of listening to the patients' story and not merely focusing on symptoms [20, 34–36]. Thus, even though the researcher has not found any other studies focusing on cognitions among frequent attenders, there are a lot of other commonalities in regards to previous research indicating that the findings could be generalized to other cultures than Swedish. Even though cultures differ, the idea that one should stay healthy and perform well is not unique for Sweden. Thus, the categories and subcategories related to those issues would be generalizable, even though different cultural contexts could contribute with nuances within them. One such nuance might be related to experiences of support from close relatives. The Swedish healthcare system has, until recently, been very generous. The austerity of today in the healthcare system does not only mean that becoming ill and on the sick list effects an individual's economy but also means that less help is available from society than there has been traditionally. That might increase the demands on relatives to support the ill person. This could be less evident in societies where there is a long tradition of involving family in care. Thus, the subcategory “being a burden to others” might not be that evident in other societies.

There are also research examples focusing on other groups that are in line with the findings in this study. For example, Andreassen et al. [37] point out a positive correlation between job stress and subjective health complaints, while Dukes Holland and Holahan [38] point out the importance of social support in relation to psychological well-being and positive health behaviours and adaption. This supports the interpretation made in this study that claims that participants who experience themselves as lonely and lack support and appreciation from others are trapped in their compensatory strategies of high performance, performances that contribute to subjective illness experiences. Even though these studies describe different populations and contexts one can, in line with the main ideas in the attachment theory [27], assume that social relations are of importance for most persons irrespective of context. On the other hand, Bergh et al. [16] found no differences in availability of social support between frequent attenders and normal users. In the light of what has been found in this study, it is interpreted that the frequent attender has a social network, but their beliefs and conditional assumptions contribute to a conception of being alone. The fact that they have a social network might also confirm that compensatory strategies work on a superficial level, keeping the person in a social context but failing in making the person conceive of him/herself as a valuable person. Living in close relationships with other persons does not protect from this; rather, it appears as the person becomes even more obsessed with his/her fear of being a burden, when he/she experiences that close ones have to care for them. Furthermore and in line with the cognitive perspective, it is most likely that a person who believes that job performance is linked to social appreciation will experience job stress to some degree.

## 5. Conclusions

The decision to attend healthcare is difficult, as the person has to confront his or her beliefs and strategies to handle suffering. It often comes when the person realises that it is impossible to stand the pain any more, and that gives rise to an urgent craving for immediate treatment and care as one must be “fighting fit” as soon as possible. Thus, in order to promote a working alliance with the patient and to be able to find out adequate interventions, it is of great importance that healthcare staff listen to the person's history, and not only examine the symptoms. Otherwise, there is a risk that healthcare staff suggest interventions that conflict with the patient's conceptions about life and how it ought to be lived. If the persons perceive that they have not been listened to, two things occur. (1) They experience themselves as even bigger failures than before. (2) They mistrust the advice given by the GP as “he/she does not have the whole story,” resulting in a lack of compliance.

In order to deal with their life situation, the frequent attender uses a high degree of what could be described as secondary control strategies [39]. These strategies take the form of conditional assumptions but do not work out very well in the long run. The participants deal with life and interact with the healthcare system out of a profound feeling of shame over their shortcomings and of themselves as inadequate persons and are constantly striving to compensate for this by intense struggling to overcome these kinds of experiences. Thus, patients' core beliefs, or self-concept, appears as important not only for health and well-being but for adjustment to life (see [40]). The compensatory strategies aim at preserving dignity and relationships with others and the participants try to keep on with them even when the strategies increase their pain and suffering. This indicates that frequent attenders need interventions that could facilitate coping with their shame and their fear of being abandoned if they fail. CBT appears as one possible approach to this [3], and considering the main theme in the findings “Struggling on my own,” a group programme could add not just new understanding of oneself and a revision of one's illness perceptions but also contribute to a sense of inclusiveness. This calls for further research, both in regards to if and how participants' beliefs about themselves, others, and the future are affected by participating in the group-treatment, and also if taking part in the group affects their frequency of attending for care.

## Figures and Tables

**Table 1:** Participant's background.

Participant	Age	Gender	Occupational status	Marital status	Health problems
A	38	Female	Sick listed	Partner	“Burnout”
B	64	Female	Working	Single	Dizziness, infections
C	25	Female	Student	Married	Undefined pain
D	30	Male	Working	Partner	Undefined pain
E	51	Female	Working	Partner	Rheumatism, asthma
F	37	Male	Working	Partner	Rheumatism
G	62	Male	Early retirement	Married	Occupational injury, migraine
H	52	Female	Unemployed	Partner	Infections, undefined pain
I	49	Male	Working	Married	Depression, venous ulcers, itching
